# Association between early serum cholinesterase activity and 30-day mortality in sepsis-3 patients: A retrospective cohort study

**DOI:** 10.1371/journal.pone.0203128

**Published:** 2018-08-30

**Authors:** Zheng-Liang Peng, Liang-Wei Huang, Jian Yin, Ke-Na Zhang, Kang Xiao, Guo-Zhong Qing

**Affiliations:** EICU, First Affiliated Hospital of the University of South China, Hengyang, Hunan, China; Heidelberg University Hospital, GERMANY

## Abstract

Low serum cholinesterase (SCHE) activity has been associated with poor prognoses in a variety of conditions, including sepsis. However, such an association has not been well characterized since the Third International Consensus Definitions Task Force modified the definition of sepsis to “life-threatening organ dysfunction due to a dysregulated host response to infection” (known as sepsis-3) in 2016. In the current retrospective cohort study, we examined whether 30-day mortality in sepsis-3 patients is associated with SCHE activity. A total of 166 sepsis-3 patients receiving treatment at an emergency intensive care unit (EICU) were included. The 30-day death rate was 33.1% (55/166). SCHE activity upon EICU admission was lower in nonsurvivors (3.3 vs. 4.5 KU/L in survivors, p = 0.0002). Subjects with low SCHE activity (defined as <4 KU/L) had higher 30-day mortality rates than subjects with normal SCHE activity (45.5%, 40/88 vs. 19.2%, 15/78; p<0.001). A multivariate logistic regression analysis revealed an association between 30-day mortality and lower SCHE activity after adjustments for relevant factors, such as acute multiple organ dysfunction. The odds ratio (OR) for every unit decrease in SCHE activity was 2.11 (95% confidence interval (CI), 1.37–3.27; p = 0.0008). The area under the curve (AUC) of SCHE activity for predicting 30-day mortality was 0.67 (95% CI 0.59–0.74), and the AUC of lactate for predicting 30-day mortality was 0.64 (95% CI 0.57–0.70). Using a combination of SCHE and lactate, the AUC was 0.74 (95% CI 0.69–0.83). These data suggest that lower SCHE activity is an independent risk factor for 30-day mortality in sepsis-3 patients.

## Introduction

Serum cholinesterase (SCHE; also known as pseudo- or butyryl-cholinesterase) is a performance metric of liver function, with lower activity reflecting more extensive liver injury [1]. Decreased SCHE activity has been associated with severity and mortality in critically ill patients [2–8], with varying prognostic values for different diseases [9]. Low SCHE activity has been correlated with systemic inflammation [10, 11] and sepsis [12–15].

The third International Consensus Definitions Task Force modified the definition of sepsis to “life-threatening organ dysfunction due to a dysregulated host response to infection” (known as sepsis-3) in 2016 [16, 17]. The modified definition included organ dysfunction as a fundamental aspect of sepsis [18]. However, whether SCHE activity is associated with short-term survival under this new definition is unclear.

The main objective of this study was to determine whether lower SCHE activity is associated with 30-day mortality independent of the Sequential Organ Failure Assessment (SOFA) score and shock status in sepsis-3 patients.

## Materials and methods

### Study design and case selection

This retrospective analysis included all patients receiving treatment for sepsis-3 in the EICU (10 beds; approximately 500 annual admissions) at the First Affiliated Hospital of the University of South China during the period from September 2014 to December 2016. This study was approved by the hospital’s Ethics Committee. Because of the anonymous and retrospective nature of this study, the ethics committee waived the need for informed consent. All of the data were fully anonymized prior to access by any of the authors.

The diagnosis of sepsis was based on the Third International Consensus Definitions for Sepsis and Septic Shock [16, 17]. Cases were excluded from the data analysis if the patient: 1) was <18 or >90 years of age; 2) was admitted to the EICU more than 10 days after the initial suspicion of sepsis; 3) had leukemia, liver cirrhosis, toxication, or HIV infection; 4) had cardiopulmonary arrest in the emergency room (ER); or 5) had a SOFA score of <2. Patient management, including volume resuscitation, the use of antibiotics and glucocorticoid steroids, sedation, blood transfusions, and mechanical ventilation, was based on the recommendations of the Surviving Sepsis Campaign that were released in 2012 [19]. SCHE activity data were not used for treatment decisions.

The case selection was carried out by 2 attending physicians independently. The initial screening was based on admission diagnoses of infection, sepsis, severe sepsis or septic shock. Then, two attending physicians independently reviewed all of the retrieved medical records to identify characteristics of the hospital admissions and to confirm the presence of infection as a reason for admission. According to the criteria of the International Sepsis Forum Consensus Conference on Definitions of Infection [20], infection was defined on the basis of the clinical presentation (for example, a febrile patient with inflammatory syndrome), laboratory findings, radiological studies and the clinical context. Cases with unconfirmed infection were excluded from the data analysis. Disputes were resolved through discussions with a chief physician. Next, two graduate students collected the patient data from the electronic health record database using EpiData 3.1. Quality assurance was carried out by having 2 independent researchers input the data into EpiData 3.1 separately. In cases of inconsistent entries, the original medical records were examined.

The primary outcome was 30-day mortality (starting from the date of EICU admission). If a patient had been discharged, information was obtained through a telephone interview.

Acute organ dysfunction was defined as an increase in the total SOFA score > = 2 or a SOFA score > = 2 in any discrete organ system, except for cardiovascular dysfunction, for which > = 1 was used [17, 21, 22]. Septic shock was defined as lactate >2 mmol/L along with either a mean arterial pressure (MAP) <65 mmHg or the use of vasoactive agents after adequate fluid resuscitation [19].

### SCHE activity

SCHE activity was determined with a commercial kit (CHE2; Roche, Mannheim, Germany) using an automatic analyzer (Roche Cobas 8000; Roche Diagnostics, Shanghai, China). SCHE activity is a routine part of liver function panels in all patients in the EICU at our institution.

### Statistical analysis

Continuous variables were presented as the means and standard deviations (SDs) or median values and interquartile ranges (IQRs), while categorical variables were presented as absolute frequencies and percentages. The Mann-Whitney and chi-squared tests were used to determine the presence of any significant difference between the means and proportions of the two groups. Spearman correlations were used to examine the associations between SCHE activity and other laboratory measurements. A generalized additive model was used to present the relationship between SCHE activity and 30-day mortality. Factors that influenced the 30-day mortality rate were identified using a multiple logistic regression analysis and were presented as odds ratios (ORs) and 95% confidence intervals (CIs). The factors entered into the regression were chosen based on their associations with short-term survival in previous studies. All lab results included in the regression analysis were based on assays conducted within 24 h after EICU admission. The normal reference range was 4–13 KU/L. SCHE activity was treated as a binary variable (low if <4.0 KU/L; normal if > = 4.0 KU/L). An ROC analysis was used to calculate the predictive power of SCHE activity.

Analyses were carried out using the R statistics (http://www.R-project.org) and EmpowerStats (http://www.empowerstats.com; X&Y Solutions, Boston, MA) programs. Statistical significance was defined as p <0.05 (2-sided).

## Results

**Fig 1:**
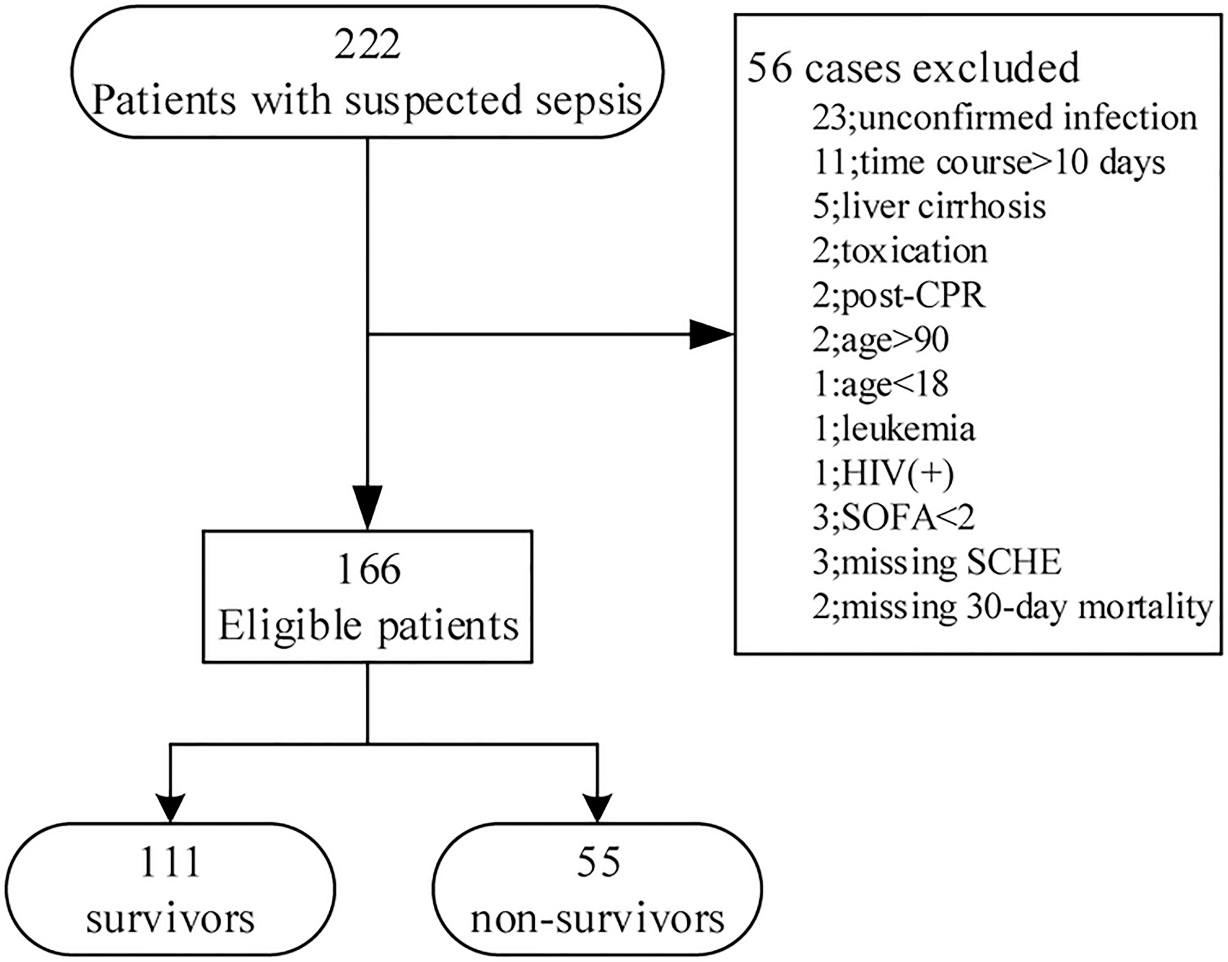
Inclusion flow diagram and outcomes for EICU sepsis-3 cohort.

The initial screen identified a total of 222 subjects (**Fig 1**). Twenty-three cases with unconfirmed infections were excluded. Eleven patients admitted to the EICU more than 10 days after the initial suspicion of sepsis were also excluded. SCHE activity was not available in 3 cases, and 30-day survival statuses could not be verified in 2 cases. After excluding the comorbid conditions listed in the exclusion criteria in the Methods section, a total of 166 patients were included in the data analysis. The 30-day mortality was 33.1% (55/166).

### Patient characteristics

The final analysis included 166 patients. A total of 57.2% (95/166) were men (**Table 1**). The mean age was 64.2 (13.7) years. The median duration from the date of the chief complaint to EICU admission was 3 days (IQR: 2–5). A total of 50.6% (84/166) of the patients had at least one chronic medical condition, e.g., diabetes mellitus (DM), coronary heart disease (CHD), chronic obstructive pulmonary disease (COPD) or hypertension. The most common sources of infection in the cohort were the lungs (34.9%), abdomen (28.3%), urinary tract (24.2%), and other or unknown infections (12.7%). The median SOFA score was 7 (IQR: 5–9), and the mean SCHE activity was 4.14 (2.02) KU/L. The SCHE activity was within the normal reference range (> = 4.0 KU/L) in 78 subjects, and below the lower limit of the normal range (<4.0 KU/L) in 88 subjects.

**Table 1:** Demographics and clinical characteristics on EICU admission by SCHE activity.

Characteristic	Total N = 166	SCHE activity > = 4(KU/L) n = 78 5.89 (1.37)	SCHE activity <4(KU/L) n = 88 2.59 (0.96)	p-value
Sex, male, n (%)	95 (57.2%)	37 (47.4%)	58 (65.9%)	0.016
Age, years, mean (SD)	64.2 (13.7)	63.4 (13.7)	64.9 (13.7)	0.418
Time course^a^ (days)	3 (2–5)	3 (1–4)	4 (2–7)	<0.001
**Source of infection, n (%)**				<0.001
Lung	58 (34.9%)	27 (34.6%)	31 (35.2%)	
Urinary tract	40 (24.1%)	28 (35.9%)	12 (13.6%)	
Abdomen	47 (28.3%)	10 (12.8%)	37 (42.1%)	
Other or unknown	21 (12.7%)	13 (16.7%)	8 (9.1%)	
**Comorbidity**^**b**^ n (%)	84 (50.6%)	41 (52.6%)	43 (48.9%)	0.634
DM n (%)	27 (16.3%)	19 (24.4%)	8 (9.1%)	0.008
CHD n (%)	18 (10.8%)	12 (15.4%)	6 (6.8%)	0.086
Hypertension n (%)	41 (24.7%)	23 (29.5%)	18 (20.5%)	0.178
COPD n (%)	32 (19.3%)	11 (14.1%)	21 (23.9%)	0.112
**Clinical data on EICU admission**				
MAP (mmHg)	81.8 (21.0)	84.2 (23.8)	79.6 (18.0)	0.406
Shock index	1.07 (0.34)	1.04 (0.35)	1.10 (0.33)	0.238
PaO2/FiO2	262.07 (200.86–348.28)	271.26 (205.17–395.69)	256.90 (197.41–338.45)	0.348
TBIL (μmol/L)	16.20 (10.83–27.05)	16.05 (11.70–26.58)	16.75 (10.00–27.33)	0.711
ALB (g/L)	29.59 (5.50)	32.81 (4.10)	26.73 (4.99)	<0.001
ALT (U/L)	28.25 (15.90–56.90)	37.70 (16.93–76.18)	23.65 (15.20–50.67)	0.133
BUN (mmol/L)	11.61 (8.12–17.08)	10.85 (7.90–14.75)	13.27 (8.83–18.31)	0.054
Cr (μmol/L)	151.25 (98.43–235.10)	150.60 (95.95–233.75)	152.15 (99.45–236.88)	0.865
Hb (g/L)	109.48 (26.15)	117.81 (26.19)	102.10 (23.93)	<0.001
PLT (10^9/L)	92.50 (50.00–163.75)	91.50 (50.25–155.75)	93.50 (49.75–164.75)	0.906
WBC (10^9/L)	13.00 (7.50–19.28)	13.40 (8.18–21.39)	12.45 (5.63–18.40)	0.311
CRP (mg/L)	132.67 (73.18–198.00)	136.00 (54.12–198.00)	129.53 (82.27–198.00)	0.768
PCT (ng/ml)	35.14 (7.05–97.33)	46.97 (7.05–100.00)	29.45 (7.28–84.35)	0.349
Lac (mmol/L)	2.79 (1.60–4.57)	2.81 (1.99–3.90)	2.65 (1.40–5.19)	0.706
Glucose (mmol/L)	7.60 (6.10–9.60)	7.60 (6.05–9.30)	7.55 (6.10–9.67)	0.997
**Severity of disease on EICU admission**				
SOFA score	7 (5–9)	6 (4–9)	7 (5–10)	0.047
Mechanical ventilation	39 (23.5%)	12 (15.4%)	27 (30.7%)	0.020
Septic shock	65 (39.2%)	31 (39.7%)	34 (38.6%)	0.884
**Treatment received within first 6 h in EICU**				
Antibiotics in first hour	144 (86.7%)	66 (84.6%)	78 (88.6%)	0.446
3 h Intravenous fluids (ml)	1550.0 (1152.5–2077.5)	1390.0 (1100.0–1983.0)	1760.0 (1210.0–2106.3)	0.058
6 h Intravenous fluids (ml)	2221.0 (1721.0–3070.3)	2136.5 (1603.3–2751.3)	2387.5 (1815.0–3147.8)	0.055
Vasoactive agent	86 (51.8%)	44 (43.6%)	42 (52.3%)	0.264
Use of sedation	42 (25.3%)	21 (26.9%)	21 (23.9%)	0.651
Use of steroids	29 (17.5%)	15 (19.2%)	14 (15.9%)	0.574
Use of albumin	58 (34.9%)	23 (29.5%)	35 (39.8%)	0.165
Blood product transfused	16 (9.6%)	7 (9.0%)	9 (10.2%)	0.785
**30-day mortality**	55 (33.1%)	15 (19.2%)	40 (45.5%)	<0.001

Continuous measures are presented as the mean (SD) or the median with the interquartile ranges (25th, 75th percentiles). Categorical variables are presented as counts and percentiles.

Abbreviations: SCHE: serum cholinesterase, EICU: emergency intensive care unit, SD: standardized difference, DM: diabetes mellitus, CHD: coronary heart disease, COPD: chronic obstructive pulmonary disease, MAP: mean arterial pressure, SOFA: Sequential Organ Failure Assessment, TBIL: total bilirubin, ALT: alanine transaminase, Hb: hemoglobin, PLT: platelet, WBC: white blood cell, CRP: C-reactive protein, PCT: procalcitonin, HR: heart rate, RR: respiratory rate, ALB: albumin, BUN: blood urea nitrogen, Cr: creatinine, Lac: lactate, OR: odds ratio.

^a^ duration from the date of the chief complaint to EICU admission

^b^ sum of the comorbidities of DM, CHD, COPD and hypertension

The male sex was over-represented in the low SCHE group (65.9% vs. 47.4%; p = 0.016). The low SCHE group also had a longer duration prior to EICU admission (4 (2–7) vs. 3 (1–4); p<0.001), a higher SOFA score (7 (5–10) vs. 6 (4–9); p = 0.016), greater use of mechanical ventilation (27 (30.7%) vs. 12 (15.4%); p = 0.020) and lower albumin (ALB) (26.73(4.99) vs. 32.81 (4.10); p<0.001) and hemoglobin (Hb) levels (102.10 (23.93) vs. 117.81 (26.19); p<0.001) than the normal group. Sources of infection were comparable between the 2 groups. Urosepsis was more common in the normal SCHE group (35.9% vs. 13.6% in the low group, p<0.001), whereas abdominal infections were more common in the low SCHE group (42.1% vs. 12.8%, p<0.001). Comorbid chronic conditions did not differ significantly between the low and normal SCHE groups, with the exception of less DM being observed in the low SCHE group (9.1% vs. 24.4%, p = 0.008). The 2 groups did not differ significantly in age, mean arterial pressure (MAP), PaO2/FiO2, shock index, total bilirubin (TBIL), creatinine (Cr), platelet (PLT), C-reactive protein (CRP), procalcitonin (PCT), lactate level (Lac) and bundle treatments (early use of antibiotics, 3 h and 6 h fluid resuscitation, etc.).

### Association between SCHE activity and 30-day mortality

SCHE activity was significantly lower in nonsurvivors in comparison to survivors (3.3 vs. 4.5 KU/L, p = 0.0002). The 30-day mortality rate was significantly higher in the low SCHE group (45.5%, 40/88 vs. 19.2%, 15/78 in the normal SCHE group). The Pearson correlation showed a significant relationship between SCHE activity and the ALB level (r = 0.70, 95% CI 0.60–0.77, p<0.0001) but not alanine transaminase (ALT) (p = 0.72) or TBIL (p = 0.71) levels.

**Fig 2:**
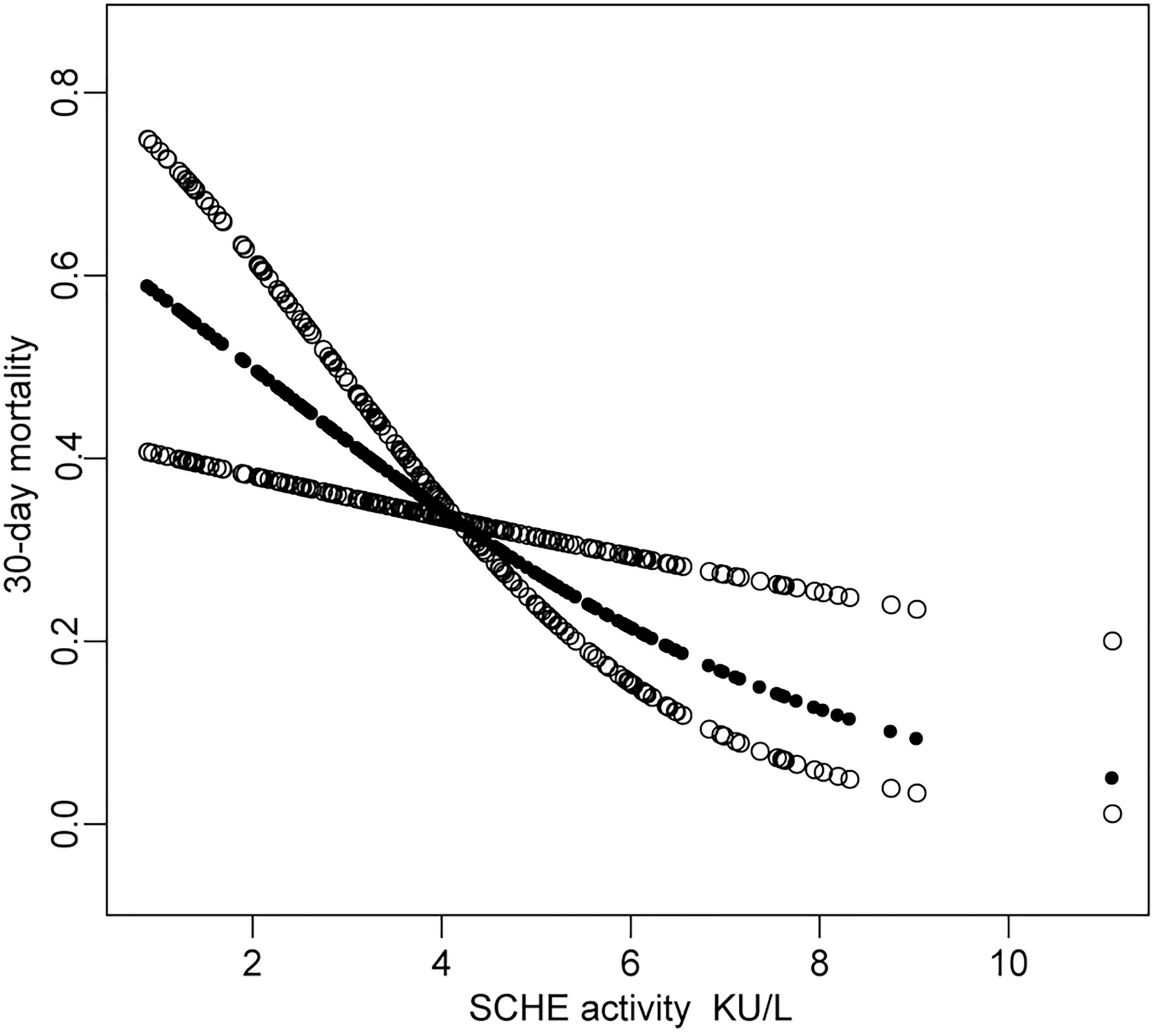
Relationship between SCHE activity and 30-day mortality. The relationship between SCHE activity and 30-day mortality with a smoothing fitting (generalized additive models (GAM)) adjusted for age and sex. In the figure, the black line in the middle indicates the estimated risk of death and the dotted lines represent the pointwise 95% CI.

Upon analysis using a generalized additive model adjusted by age and sex, SCHE activity was inversely correlated with 30-day mortality (**Fig 2**).

A univariate logistic regression indicated that 30-day mortality is associated with the following factors (**Table 2**): advanced age (OR 1.03, 95% CI 1.00–1.06, p = 0.0227), lower SCHE activity (OR 1.41, 95% CI 1.16–1.70, p = 0.0004), higher lactate levels (OR 1.24, 95% CI 1.11–1.39, p = 0.0002), higher SOFA scores (OR 1.12, 95% CI 1.01–1.24, p = 0.035), higher BUN levels (OR 1.06, 95% CI 1.02–1.11, p = 0.0041), and Glasgow Coma Scale scores <13 (OR 4.98, 95% CI 2.29–10.81, p<0.0001). The 30-day mortality rate was 12.5% (5/40) for infections originating from the urinary tract, 43.1% (25/58) for those from the lungs, 34.0% (16/47) for those from the abdomen, and 42.9% (9/21) for those from other and unknown sources. In subgroup analyses of different infection sources (**S1 Table**), lower SCHE activity was associated with increased 30-day mortality in all subgroups.

**Table 2:** The univariate logistic regression analyses of the variables for 30-day mortality.

variables	OR	95% CI	p-value
**Age**, years	1.03	(1.00, 1.06)	0.0227
**Sex**, male	1.33	(0.69, 2.57)	0.4008
**SCHE activity** (×-1) (KU/L)	1.41	(1.16, 1.70)	0.0004
**Lac** (mmol/L)	1.24	(1.11, 1.39)	0.0002
**SOFA score**	1.12	(1.01, 1.24)	0.0346
**PaO2/FiO2 <300**	2.35	(1.17, 4.74)	0.0169
**ALB** (g/L)	0.93	(0.88, 0.99)	0.0251
**BUN** (mmol/L)	1.06	(1.02, 1.11)	0.0041
**Cr** (μmol/L)	1.00	(1.00, 1.01)	0.0736
**Glasgow Coma Scale <13**	4.98	(2.29, 10.81)	<0.0001
**Source of infection**			
Urinary tract	1		
Lung	5.30	(1.82, 15.48)	0.0023
Abdomen	3.61	(1.19, 11.01)	0.0239
Other or unknown	5.25	(1.47, 18.78)	0.0108

Data are odds ratios (95% CI). We calculated odds ratios for 30-day mortality using univariate logistic regression analyses. The total number of participants was the same for all of the models.

Abbreviations: SCHE: serum cholinesterase, SOFA: Sequential Organ Failure Assessment, ALB: albumin, BUN: blood urea nitrogen, Cr: creatinine, Lac: lactate, OR: odds ratio.

Lower SCHE activity was significantly associated with increased 30-day mortality in discrete organ dysfunction subgroups (**Table 3**), with the exception of central nervous system dysfunction (OR 1.07, 95% CI 0.73–1.57, p = 0.722). There were no significant subgroup interactions.

**Table 3:** Logistic regression analyses evaluating the influence of lower SCHE activity on 30-day mortality in the acute organ dysfunction subgroups.

Acute organ dysfunction criteria	N	OR (95% CI)	p*-*value	p for interaction
Cardiovascular dysfunction				0.2280
MAP > = 70 mmHg	113	1.33 (1.07, 1.65)	0.0089	
MAP <70 mmHg	53	1.79 (1.13, 2.84)	0.0130	
Respiratory dysfunction				0.2502
PaO2/FIO2 > = 300	67	1.70 (1.13, 2.55)	0.0104	
PaO2/FIO2 <300	99	1.31 (1.06, 1.62)	0.0137	
Hepatic dysfunction				
Total bilirubin <33 μmol/L	133	1.36 (1.10, 1.69)	0.0039	0.6030
Total bilirubin > = 33 μmol/L	33	1.55 (1.00, 2.38)	0.0476	
ALT <41 U/L	98	1.37 (1.07, 1.76)	0.0124	0.6535
ALT > = 41 U/L	68	1.51 (1.10, 2.07)	0.0111	
ALB >30	80	1.39 (1.03, 1.87)	0.0307	0.5185
ALB < = 30	86	1.61 (1.15, 2.26)	0.0056	
Renal dysfunction				0.6899
Creatinine <171 μmol/L	103	1.45 (1.12, 1.87)	0.0048	
Creatinine > = 171 μmol/L	63	1.34 (1.01, 1.78)	0.0453	
Central nervous system dysfunction				0.1194
Glasgow Coma Scale > = 13	129	1.54 (1.20, 1.97)	0.0007	
Glasgow Coma Scale <13	37	1.07 (0.73, 1.57)	0.7220	
Hematologic dysfunction				0.8895
Platelets > = 100 (10^9/L)	76	1.41 (1.10, 1.80)	0.0059	
Platelets <100 (10^9/L)	90	1.45 (1.06, 1.97)	0.0184	
Hypoperfusion				0.3354
Lactate < = 2 mmol/L	57	1.71 (1.12, 2.62)	0.0135	
Lactate >2 mmol/L	109	1.36 (1.09, 1.70)	0.0066	
Septic shock				0.8583
no shock	101	1.39 (1.10, 1.75)	0.0052	
shock	65	1.44 (1.03, 2.01)	0.0315	

Lower SCHE activity was significantly associated with increased 30-day mortality in discrete acute organ dysfunction subgroups, with the exception of central nervous system dysfunction. Abbreviations: SCHE: serum cholinesterase, MAP: mean arterial pressure, ALT: alanine transaminase, ALB: albumin, OR: odds ratio.

After adjusting for other factors, lower SCHE activity remained significantly associated with 30-day mortality (**Table 4**). For every unit (KU/L) decrease in SCHE activity, the OR for 30-day mortality was 2.11 (95% CI 1.37–3.27) in the final adjusted model. After adjusting for potential confounding variables, lower SCHE activity remained a risk factor for 30-day mortality in both the septic shock and no shock subgroups (**Table 5**). In a series of subgroup analyses based on other contributing factors (**S1 Table**), lower SCHE activity was inversely associated with 30-day mortality in all subgroups. There were no significant subgroup interactions.

**Table 4:** Associations between lower SCHE activity and 30-day mortality in sepsis-3 patients.

	OR	95% CI	p-value
Model 1	1.41	(1.16, 1.70)	0.0004
Model 2	1.39	(1.14, 1.70)	0.0010
Model 3	1.83	(1.26, 2.65)	0.0015
Model 4	2.04	(1.33, 3.13)	0.0011
Model 5	2.11	(1.37, 3.27)	0.0008

Model 1: unadjusted; model 2: adjusted for age and sex; model 3: adjusted for age, sex; albumin; C-reactive protein; time course; comorbidity; source of sepsis; hemoglobin; SOFA score; model 4: adjusted for model 3 plus cardiovascular dysfunction, respiratory dysfunction, hepatic dysfunction, renal dysfunction, central nervous system dysfunction, hematologic dysfunction, lactate >2 mmol/L, septic shock; model 5 adjusted for model 4 plus antibiotics in first hour, 3 h intravenous fluids (ml), 6 h intravenous fluids (ml).

**Table 5:** Associations between lower SCHE activity and 30-day mortality in septic shock and no shock subgroups.

Model	No shock model (n = 101)	Septic shock model (n = 65)
	OR (95% CI) p	OR (95% CI) p
Nonadjusted	1.39 (1.10, 1.75) 0.0052	1.44 (1.03, 2.01) 0.0315
Model 1	1.38 (1.08, 1.75) 0.0091	1.40 (0.99, 1.98) 0.0576
Model 2	1.73 (1.06, 2.82) 0.0294	2.31 (1.17, 4.55) 0.0156

Model 1 adjusted for age and sex; model 2 adjusted for age, sex, albumin, C-reactive protein, time course, comorbidity, source of sepsis, hemoglobin and SOFA score.

### Value of early SCHE activity for predicting 30-day mortality

**Fig 3:**
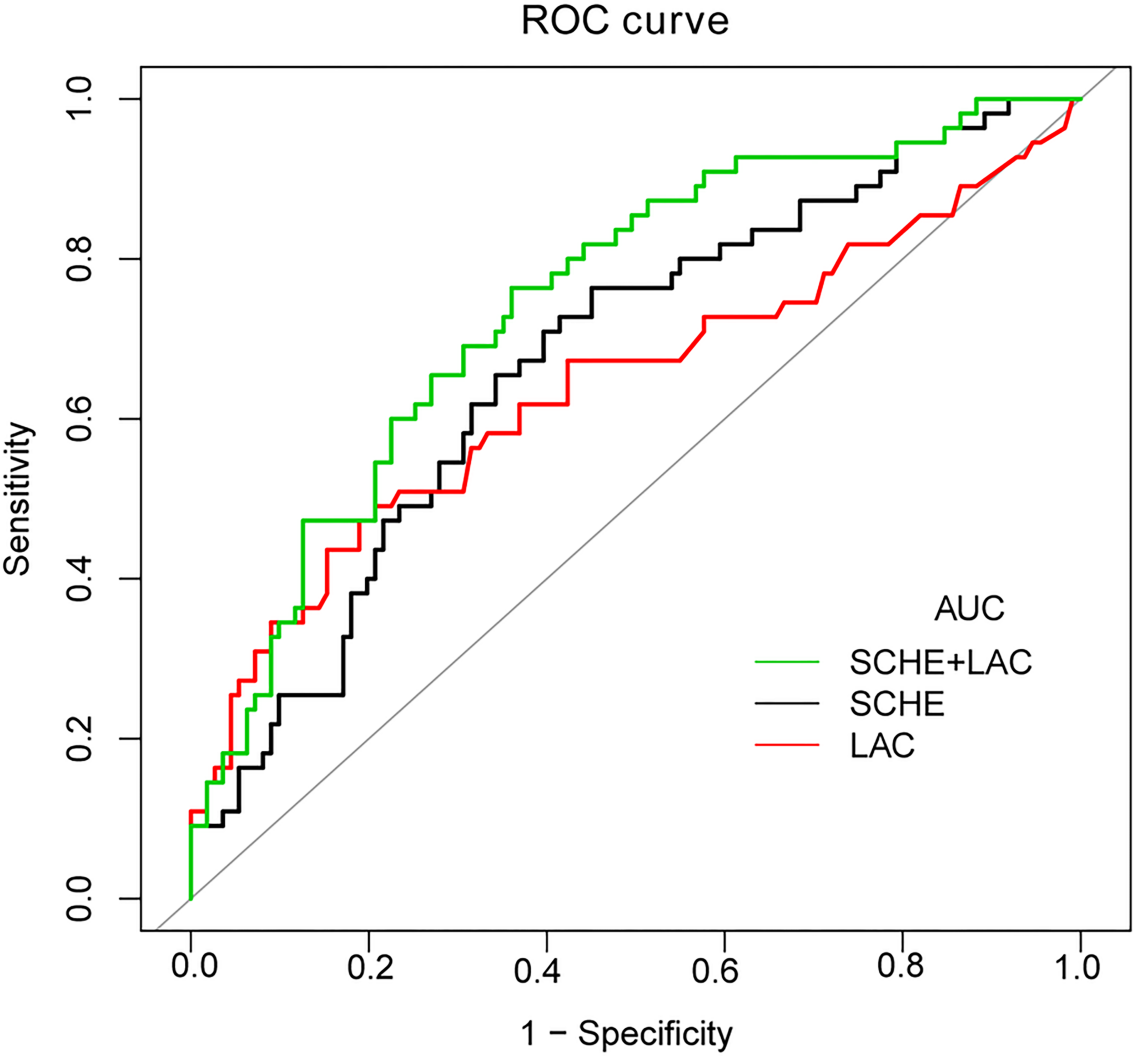
ROC analysis for predicting 30-day mortality in sepsis-3 patients. AUCs of various parameters for predicting 30-day mortality in patients with sepsis-3. Using a combination of SCHE and Lac, the AUC was 0.74 (95% CI 0.69–0.83), which was better than Lac alone (p = 0.0046). AUC, area under the curve; SCHE, serum cholinesterase activity; Lac, lactate level.

An ROC curve analysis revealed that the AUC of SCHE activity for predicting 30-day mortality was 0.67 (95% CI 0.59–0.74) (**Table 6**). The optimal cut-off value was 4.26 KU/L (lower limit of the normal reference range: 4 KU/L). At a clinically pragmatic level of 4 KU/L, the sensitivity and specificity of using SCHE activity to predict 30-day mortality were 0.73 (95% CI 0.59–0.84) and 0.57 (95% CI 0.47–0.66), respectively. The AUC for lactate was 0.64 (95% CI 0.57–0.70). At an optimal cut-off of 3.88 mmol/L, the sensitivity and specificity of using lactate to predict 30-day mortality were 0.49 (95% CI 0.35–0.63), and 0.79 (95% CI 0.71–0.86), respectively. When using a combination of SCHE and lactate, the AUC was 0.74 (95% CI 0.69–0.83), which was better than lactate alone (p = 0.0046) (**Fig 3**).

**Table 6:** ROC analysis of variables for predicting 30-day mortality in sepsis-3 patients.

For prediction of 30-day mortality	SCHE (KU/L)	Lac (mmol/L)	SCHE+Lac
Optimal cutoff	4.26	3.88	
AUC, (95% CI)	0.67 (0.59–0.74)	0.64 (0.57–0.70)	0.74 (0.66–0.82)
Sensitivity, (95% CI)	0.76 (0.63–0.87)	0.49 (0.35–0.63)	0.82 (0.69–0.91)
Specificity, (95% CI)	0.55 (0.45–0.64)	0.79 (0.71–0.86)	0.55 (0.45–0.64)

AUC, area under the curve; SCHE, serum cholinesterase activity; Lac, lactate level.

## Discussion

In the current study, we demonstrated that lower SCHE activity in sepsis-3 patients is associated with increased 30-day mortality in an EICU setting, even after adjusting for potential confounding factors, including acute multiple organ dysfunction. For every unit (KU/L) decrease in SCHE activity upon EICU admission, the risk of 30-day mortality increased by 2.11 (95% CI 1.37–3.27). An ROC analysis showed a moderate performance of SCHE activity in predicting 30-day mortality, at 0.67 (95% CI 0.59–0.74). The AUC of serum lactate was 0.64 (95% CI 0.57–0.70). A combination of SCHE and lactate enhanced the performance to 0.74 (95% CI 0.69–0.83). These findings suggest that early SCHE activity is a useful biomarker for predicting short-term survival in sepsis-3 patients.

We observed an association between SCHE activity and albumin levels (r = 0.70, p<0.0001). Despite this finding, lower SCHE activity was associated with 30-day mortality independent of acute liver dysfunction in our analysis. In subgroup analyses based on factors that influenced 30-day mortality, the association between SCHE activity and 30-day mortality remained robust in all subsets of liver dysfunction. These findings suggest that the SCHE concentration is independent of acute liver dysfunction in the early phase of sepsis-3. As a result, SCHE activity is considered by some to be an inaccurate measure of liver function [23]. The mechanisms that underlie SCHE reduction in sepsis-3 have not been fully elucidated. SCHE activity is affected by acute infection and inflammatory processes [23, 24]. Some hypotheses for this have been postulated [15]. The first is that, along with the progression of the disease, liver dysfunction leads to reduced synthesis of SCHE. The second is that an increase in capillary permeability is responsible for transcapillary loss of SCHE. The third is related to the dilution effect of fluid challenges. The last has to do with the increased catabolism of SCHE and the inhibition of SCHE by inflammatory mediators (cytokines). In general, we primarily considered two possibilities. One was that with the progression of sepsis, systemic inflammation affecting the activity is triggered, and there is no change in the amounts of SCHE. The other is that the decrease in SCHE levels leads to low activity because of excessive consumption and/or restrained supplementation. In short, specific mechanisms need to be further studied. “SCHE is, in many ways, an enigma. It appears that while SCHE is far from being vestigial or nonfunctional, its functions are shadowy and poorly understood in comparison to the brilliance and definition of acetylcholinesterase (AChE)”, Glynis Johnson commented [25]. An analysis of the published literature suggests that SCHE has some principal functions: detoxification, acetylcholine hydrolysis, fat metabolism and scavenging of polyproline-rich peptides [26]. These functions may have potential effects on the prognosis of sepsis. Our study suggests that SCHE activity corresponds to the protein-energy malnutrition variables (ALB, HB) that reflect the response to systemic inflammation. These findings are generally consistent with previous studies showing an association between low SCHE activity and anemia and hypoalbuminemia [8, 10, 15].

In the recently modified definition of sepsis (sepsis-3), acute organ dysfunction is highlighted. In the current study, lower SCHE activity remained significantly associated with 30-day mortality even after adjusting for acute multiple organ dysfunction. Increasing evidence suggests that the cholinergic anti-inflammatory pathway plays a critical role in the systemic response to infection [27–29]. The fact that parasympathetic nervous system activity could influence circulating cytokines [30, 31] helps to explain the reduction in SCHE activity in sepsis. The findings from the current study in sepsis-3 patients are generally consistent with observed associations between SCHE and short-term mortality [13, 32]. The present study also found that the association between SCHE activity and 30-day mortality was modified in the presence of central nervous system dysfunction. We speculate that sympathetic/parasympathetic nervous system activity plays an important role in the SCHE pathway. A previous study [6] in patients with traumatic brain injury (TBI) showed that although SCHE activity on admission was differentially reduced according to the severity of the injury, infection status and outcome, SCHE activity was not an independent predictor of 90-day mortality after adjusting for age and gender. The association between SCHE activity and short-term mortality in the subgroup of central nervous system dysfunction needs to be investigated further.

Blood lactate has been associated with poor prognoses in patients with sepsis [33]. Lactate was a low-sensitivity and high-specificity test for 30-day mortality. The analysis in our study showed a higher sensitivity of SCHE activity for predicting 30-day mortality than the lactate level, and, thus, it could be an effective screening test for short-term mortality in sepsis-3 patients.

There are several limitations to our study. The current study is retrospective by nature and thus was subjected to bias due to confounding factors. Furthermore, we are unable to illuminate the mechanisms behind our findings. The study is also limited by its small sample size. As a single-center study, the results must be interpreted with caution when extrapolating them into other settings. The primary outcome of the study only included 30-day mortality. The study did not collect data regarding long-term outcomes and did not investigate the relationship between the changes in SCHE activity and the prognosis. Despite these limitations, in our opinion, the association between lower SCHE activity and short-term mortality in sepsis-3 patients is valid.

## Conclusions

The current study confirmed an association between early SCHE activity and 30-day mortality, independent of SOFA scores and shock status, in sepsis-3 patients.

## Supporting information

S1 Table Association between lower SCHE activity and 30-day mortality by subgroups of selected risk factors. (DOCX)

S2 Table STROBE_checklist_v4_combined_PlosMedicine. (DOCX)

S1 Fig Raw clinical data_10.1371/journal.pone.0203128. (XLSX)
