# Supplementary material for: Association between early serum cholinesterase activity and 30-day mortality in sepsis-3 patients: A retrospective cohort study
Source: PLoS One. 2018 Aug 30;13(8):e0203128. doi: 10.1371/journal.pone.0203128 (PMC6117034; doi:10.1371/journal.pone.0203128)
Supplement: S1 Table — (DOCX) [file pone.0203128.s001.docx]

**S1 Table. Association between lower SCHE activity and 30-day mortality by subgroups of selected risk factors**

| **Subgroup** | **n** | **OR** | **95% CI** | **p** | **p**  **for interaction** |
| --- | --- | --- | --- | --- | --- |
| SEX |  |  |  |  | 0.4106 |
| female | 71 | 1.56 | (1.12, 2.18) | 0.009 |  |
| male | 95 | 1.32 | (1.04, 1.67) | 0.021 |  |
| AGE (years) |  |  |  |  | 0.3369 |
| 24 - 60 | 51 | 1.84 | (1.16, 2.90) | 0.009 |  |
| 61 - 69 | 58 | 1.25 | (0.90, 1.73) | 0.177 |  |
| 70 - 88 | 57 | 1.32 | (0.99, 1.76) | 0.060 |  |
| HB (g/L) |  |  |  |  | 0.5897 |
| 43 - 96 | 55 | 1.82 | (1.18, 2.80) | 0.007 |  |
| 97 - 118 | 54 | 1.40 | (0.95, 2.05) | 0.085 |  |
| 120 - 196 | 57 | 1.42 | (1.02, 1.96) | 0.036 |  |
| BUN (mmol/L) |  |  |  |  | 0.9865 |
| 2.1 - 8.96 | 55 | 1.38 | (0.97, 1.97) | 0.076 |  |
| 9 - 14.77 | 55 | 1.41 | (0.98, 2.01) | 0.062 |  |
| 14.8 - 40.2 | 56 | 1.35 | (0.99, 1.84) | 0.057 |  |
| CRP (mg/L) |  |  |  |  | 0.3781 |
| 0.5 - 96.5 | 55 | 1.26 | (0.96, 1.65) | 0.102 |  |
| 96.8 - 180.0 | 55 | 1.46 | (1.04, 2.05) | 0.030 |  |
| 180.5 - 198 | 56 | 1.81 | (1.15, 2.84) | 0.010 |  |
| PCT (ng/ml) |  |  |  |  | 0.8369 |
| 0.01 - 14.4 | 55 | 1.35 | (0.99, 1.84) | 0.061 |  |
| 14.5 - 67.5 | 55 | 1.55 | (1.05, 2.29) | 0.027 |  |
| 67.6 - 100 | 56 | 1.49 | (1.05, 2.12) | 0.025 |  |
| TBIL (μmol/L) |  |  |  |  | 0.8557 |
| <=20 | 104 | 1.39 | (1.09, 1.76) | 0.007 |  |
| >20 | 62 | 1.44 | (1.05, 1.98) | 0.02 |  |
| ALT (U/L) |  |  |  |  | 0.6535 |
| <=41 | 98 | 1.37 | (1.07, 1.76) | 0.012 |  |
| >41 | 68 | 1.51 | (1.10, 2.07) | 0.011 |  |
| ALB (g/L) |  |  |  |  | 0.5185 |
| >30 | 80 | 1.39 | (1.03, 1.87) | 0.031 |  |
| <=30 | 86 | 1.61 | (1.15, 2.26) | 0.006 |  |
| Shock index |  |  |  |  | 0.8688 |
| <=1 | 80 | 1.42 | (1.07, 1.90) | 0.017 |  |
| >1 | 86 | 1.38 | (1.07, 1.77) | 0.013 |  |
| Source of infection |  |  |  |  |  |
| *Lung* | 58 | 1.35 | (1.00, 1.82) | 0.050 |  |
| *Urinary tract* | 40 | 1.39 | (0.80, 2.41) | 0.240 |  |
| *Abdomen* | 27 | 1.72 | (1.06, 2.78) | 0.027 |  |
| *Other or unknown* | 21 | 1.44 | (0.87, 2.38) | 0.152 |  |
| DM | 27 | 1.19 | (0.68, 2.08) | 0.542 |  |

We analyzed lower SCHE activity in association with 30-day mortality across subgroups of various risk factors. We detected no significant subgroup interactions.

Abbreviations: SCHE: serum cholinesterase, DM: diabetes mellitus, TBIL: total bilirubin, ALT: Alanine transaminase, Hb: hemoglobin, PLT: platelet, CRP: C-reactive protein, PCT: procalcitonin, ALB: albumin, BUN: blood urea nitrogen, Cr: creatinine.
